# Improvement in Menopause-Associated Hepatic Lipid Metabolic Disorders by Herbal Formula HPC03 on Ovariectomized Rats

**DOI:** 10.1155/2020/1409376

**Published:** 2020-07-19

**Authors:** BoYoon Chang, Dae Sung Kim, SungYeon Kim

**Affiliations:** ^1^Institute of Pharmaceutical Research and Development College of Pharmacy, Wonkwang University, Iksan, Jeonbuk, Republic of Korea; ^2^Hanpoong Pharm. Co., Ltd., Jeonju-si, Jeonbuk, Republic of Korea

## Abstract

Postmenopausal women have an increased risk of developing nonalcoholic fatty liver disease (NAFLD). We formulated a combination of three herb mixtures (HPC03) and observed lipid-lowering efficacy. HepG2 cells were treated with oleic acid to induce an NAFLD model (in vitro). Also, we investigated potential of HPC03 in an ovariectomize- (OVX-) induced NAFLD model (in vivo). We separated the mice into six groups, as follows: SHAM, OVX, OVX + *β*-estradiol, and OVX + HPC03 (50, 100, and 200 mg/kg). Rats were administered with/without HPC03 for 12 weeks. HPC03 dose dependently inhibited the lipid accumulation involved in lipogenesis in HepG2 cells. The body weight, fat mass, and weights of the liver were decreased in the OVX group than that in the other groups. HPC03 had decreased adiposity that was induced by OVX. HPC03 treatment reduced liver lipid deposition and prevented the increase in serum and liver triglyceride export when there was a deficiency in estradiol. HPC03 improves OVX-induced fatty liver and lipid metabolism. These findings suggest that HPC03 from postmenopausal women has a protective effect during NAFLD conditions.

## 1. Introduction

Nonalcoholic fatty liver disease (NAFLD) is a broad concept that includes an excessive accumulation of triglyceride in the liver, from nonalcoholic steatohepatitis and cirrhosis [1–3]. For an accurate definition of nonalcoholic fats, secondary causes such as heavy alcohol intake or drugs that induce fat accumulation in the liver must be eliminated. In the earliest stages of nonalcoholic fatty liver disease, the triglyceride content among the healthy and dry people with simple lipidosis falls into the top 95% or more and intracellular defined when the proportion of neutral fat particles is 5% or more [4]. The prevalence of nonalcoholic fatty liver was reported to increase in men in their 30 s–40 s and then decreased, while women tended to increase sharply in their 60 s after menopause. Menopausal women experience significant changes in hormones due to a decrease in the female hormone estrogen. Postmenopausal changes in female hormones cause various symptoms such as osteoporosis and cardiovascular disease, thereby changing the quality of life [2, 5–8]. In menopause, estrogen is decreased specifically, cholesterol is accumulated in the body, and fatty liver is induced. Due to the effects of these hormones, women are more likely to be exposed to fatty liver than men [9, 10].

The progression of NAFLD is mainly due to abnormal lipid accumulation, and suppressing lipid accumulation is the most important point for NAFLD drug developers [11, 12]. Currently, metformin, statins, fibrates, etc. are used in clinical practice. However, these drugs are known for serious side effects, including osteoporosis. Therefore, the search for new therapeutic candidates for the treatment of NAFLD is urgent [13, 14].

There are an increasing number of studies currently focusing on herbal extracts and natural products, and many of these studies have found herbal products with the powerful effects of NAFLD [13, 15]. Some herbal plants, such as *Panax ginseng* [12], *Camellia oleifera* [16], *Gynostemma pentaphyllum* [17, 18], *Rhizophora mangle L.* [19], *Syzygium simile* [20], and *elica gigas*, have a potent effect on nonalcoholic fatty liver symptoms. In our previous study, HPC03 (mixture of *Angelica gigas, Cnidium officinale Makino*, and *Cinnamomum cassia Presl*) was effective in treating osteoporosis with estrogen-like action. With this in mind, we tried to test the therapeutic effect of HPC03 on fatty liver induced by menopause. This study aimed to find out whether the HPC03 inhibits the hepatic lipotoxicity model of HepG2 cell steatosis and nonalcoholic fatty liver disease (NAFLD) in female ovariectomized (OVX) rats.

## 2. Materials and Methods

### 2.1. Plant Material and Extraction

HPC03 was purchased from the Hanpoong Pharm. Co., Ltd. HPC03 was extracted in the same way as in the study by Chang et al. [21]. For standardization of the HPC03, decursin, ferulic acid, or cinnamic acid were used as the marker compositions. HPC03 contained the contents of decursin (121.79 ± 0.3 mg/g), ferulic acid (0.73 ± 0.1 mg/g), or cinnamic acid (0.29 ± 0.1 mg/g).

### 2.2. Cell Culture

We purchased HepG2 cells from the ATCC (American Type Culture Collection, Manassas, VA, USA). Cells were cultured in DMEM media with 10% fetal bovine serum and 5% antibiotic added using an incubator controlled at 5% CO_2_ at 37°C. Nonfat BSA conjugated oleic acid was used to establish a lipid accumulation model using HepG2 cells.

### 2.3. Cytotoxicity

HepG2 cells were seeded in 24-well plates at 5 × 10^4^ cells/well, and when the cell grown to 70% (up to 12 hr), HPC03 was treated at a concentration of 3, 10, 30, 100, or 300 *μ*g/mL. After 24 hours, the experiment was terminated and cell viability was confirmed using the MTT reagent.

### 2.4. FFA-Induced Lipid Accumulation in HepG2 Cells

After reaching approximately 70% confluence, they were treated in the presence or absence of 200 *μΜ* oleic acid, with HPC03 at 3, 10, 30, or 100 *μ*g/mL. After the experiment, the cells were washed with PBS and fixed with 4% paraformaldehyde for 15 min at room temperature. Cells were washed with PBS, incubated with 60% isopropanol for 5 min, and then stained with 0.1% oil red o staining solution for 1 h. After the oil red *o* deposited on the fat was observed under a microscope, the absorbance (510 nm) was measured by dissolving the red stained in lipid with isopropanol for 10 min to quantify the amount of lipid.

### 2.5. Animals

6-week-old female Sprague-Dawley (SD) rats were provided by Orient Bio. Animals were divided into two for each cage; the temperature in the room is adjusted to 23 ± 2°C, and the humidity is adjusted to 55–60%. The AIN 76A diet was fed so that there was no effect of supplying estrogen. After an acclimatization period of one week, the rats were anesthetized with 2% isoflurane, and then operated group (SHAM) and ovariectomized operation (OVX) were performed. The study was approved by the Wonkwang University Animal Care Committee (WKU16-07). One week after the completion of surgery, DW was orally administered to the SHAM group, and the OVX group was divided into 5 groups: DW, HPC03 50, 100, and 200 mg/kg (p.o.), or estradiol 10 *μ*g/kg (i.p.) was injected for 12 consecutive weeks. Body weight was measured weekly until all experiments were completed. Abdominal fat and bone density measurements were measured one week before the end of the experiment with pDEXA (InAlyzer, Medikors, Korea). On the day of the end of the experiment, animals were anesthetized with ethyl ether; then, blood was sampled from the abdominal aortic artery, and the liver was immediately weighed. Serum obtained after centrifugation and liver tissue were stored at −80 until use.

### 2.6. Estimation of Biochemical Parameters

Serum from the collected blood samples was used to measure aspartate aminotransferase (AST) and alanine aminotransferase (ALT) levels according to the method described by Reitman and Frankel Reitman. Serum triglyceride (TG) and total cholesterol (TC) levels were estimated enzymatically by commercially available kits (TG: Biovision Co., USA; TC: Asan Pharm Co., Korea).

### 2.7. Estimation of Lipid Contents in Liver

According to the Folch et al. method [22], triglyceride (TG) and total cholesterol (TC) levels in liver tissues were estimated. In brief, liver tissues were homogenized in cold KCl-tris buffer. The homogenate was extracted with a mixture of chloroform and methanol (2 : 1, v/v), and the mixture was centrifuged at 3,000 rpm for 15 min. The organic layer was collected and dried, and the residue was dissolved in isopropanol. We estimated TG and TC levels enzymatically by commercially available kits (TG: Biovision Co., USA; TC: Asan Pharm Co., Korea). The results of TG and TC quantified the amount of protein in liver tissue used in the experiment and expressed as mg/g liver.

### 2.8. Measurement of Liver MDA

According to the Ohkawa et al. method [23], MDA levels in liver tissues were estimated. A sample was prepared by adding 9 times the volume of extracted liver tissue to KCl (pH 7.4) and then homogenized. The homogenized sample was mixed with TBA, SDS, acetic acid, and DW and boiled at 100°C for 60 min. After all the reaction was over, *n*-butanol was added, mixed, and then centrifuged to collect the upper layer. A calibration curve, consisting of accurately prepared standard MDA solutions (from 2 to 20 nmol/mL), was also run for quantitation. Measurements were done in triplicate. MDA levels were expressed as *μ*mol/g liver.

### 2.9. Measurement of Liver GSH Levels

GSH in liver tissue was assayed by the method of Griffith in 1979 [24]. In brief, 100 *μ*l of tissue supernatant was placed in a 3 ml cuvette; 750 *μ*l of 10 mM 5–5′-dithio-bis-2-nitrobenzoic acid (DTNB) was added, and the mixture was incubated for 3 min at room temperature. Then, 150 *μ*l of 1.47 mM *β*-NADPH was added and mixed rapidly by inversion, and the rate of 5-thio-2-nitrobenzoic acid was measured spectrophotometrically for 1 min at 412 nm. The results were expressed as *μ*mol/g liver.

### 2.10. Histological Analysis

Portions of the frozen or 10% formalin-fixed liver tissues were embedded in FCS22 R Frozen Section Media (Leica, Richmond, IL, United States) and then sectioned at 10 *μ*m thickness using a cryomicrotome (Leica CM1860, Leica Biosystem, Nussloch, Germany) and a rotary microtome (Leica RM2235, Leica Biosystem, Nussloch, Germany), respectively. Sections were fixed on silicon-coated glass slides (Microslides, Muto Pure Chemical Co. Ltd., Tokyo, Japan). And then, the frozen tissues were stained with oil red o (Sigma-Aldrich, St. Louis, MO, USA) and lightly counterstained with hematoxylin. The sections were examined under an inverted microscope (LeicaDMI6000 B, Leica Microsystems CMS GmbH, Wetzlar, Germany), and the images were captured.

### 2.11. Statistical Analysis

All data are expressed as average ± standard deviation. Student's *t*-test was used to compare significant differences between groups. Statistical significance was defined as *p* < 0.05. All statistical analyses were done using GraphPad Prism 5.0 software (Chicago, IL, USA).

## 3. Results

### 3.1. Effect of HPC03 on Cytotoxicity

**Figure 1:**
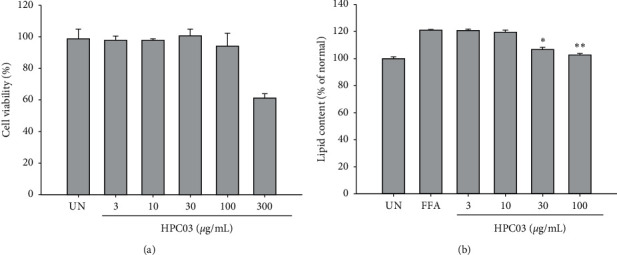
Effect of HPC03 on cell viability and FFA-induced lipid accumulation in HepG2 cells. (a) Cells were treated with HPC03 and subsequently incubated for 24 h. Cell viability was assessed as described in Materials and Methods. (b) FFA-induced lipid accumulation was significantly inhibited by HPC03. HepG2 cells treated with HPC03, in the presence or absence of FFA, were stained with an Oil Red O solution. The results are presented as mean ± SD. Significant differences compared with the untreated group (UN) or FFA‐induced group (FFA) are indicated by ^*∗*^*p* < 0.05 and ^*∗∗*^*p* < 0.01.

HPC03 treatment for 24 h with concentrations of up to 100 *μ*g/mL showed no significant effects on HepG2 cell viability (Figure 1(a)). Higher doses (300 *μ*g/mL) of HPC03 reduced cell viability up to 61.9 ± 3.09%

### 3.2. Effect of HPC03 on FFA-Induced Lipid Accumulation in HepG2 Cells

Supplementation with FFA significantly induced lipid accumulation in HepG2 cells. In the group treated with only FFA, the staining amount of oil red *o* was significantly increased compared to BSA-only treated cells. Lipid accumulation in HepG2 cells was induced, and oil red *o* content was significantly and concentration dependently decreased in the HPC03-treated group in Figure 1(b).

### 3.3. Effect of HPC03 on Food Intake, Body, and Organ Weight

The overall daily food intake was increased in the OVX group more than that in the SHAM group. Food intake did not differ between groups (Table 1). Initial body weights were similar among all groups. In all groups, body weight increased over time; final body weight was higher (*p* < 0.01) in the OVX group than that in the SHAM group.

Total body weight gain was higher in the OVX group than that in the SHAM group. OVX + *E*2 inhibited the increment of OVX-induced weight gain and abdominal fat. OVX + HPC03 200 mg/kg inhibited the increment of OVX-induced weight gain. Increases in abdominal fat in the OVX group were significantly attenuated in the OVX + HPC group. Liver weight loss was observed by OVX, but liver weight change by HPC03 and E2 treatment was not observed.

### 3.4. Effect of HPC03 Serum Transaminase Activities and Lipid Profiles

The effects of ovariectomy or HPC03 treatment on biochemical serum variables are presented in Table 2. The OVX groups showed a 35%p and 40%p increase in serum ALT and AST, respectively, compared to the SHAM group. Treatment with 50, 100, and 200 mg/kg in the OVX + HPC03 group significantly decreased the ALT levels (6.8%p, 10.5%p, and 42.0%p, respectively) compared to the ALT levels of the OVX group. The 200 mg/kg OVX + HPC03 group had significantly decreased AST levels (18.4%p) compared to the AST levels of the OVX group. Following 12 weeks of treatment, the TG and TC levels were significantly elevated, by 122.1%p and 50.9%p, respectively, in the OVX group compared to those of the SHAM group. In the 100 and 200 mg/kg OVX + HPC03 group, the TG levels were significantly lower, by 27.8%p and 31.8%p, respectively, compared to those of the OVX group (*p* < 0.01). However, no significant change in serum total cholesterol was observed among the treated groups. In contrast, the OVX + *E*2 group had significantly lower ALT and serum TG levels compared to the OVX group.

### 3.5. Effect of HPC03 Hepatic Lipid Profiles

**Figure 2:**
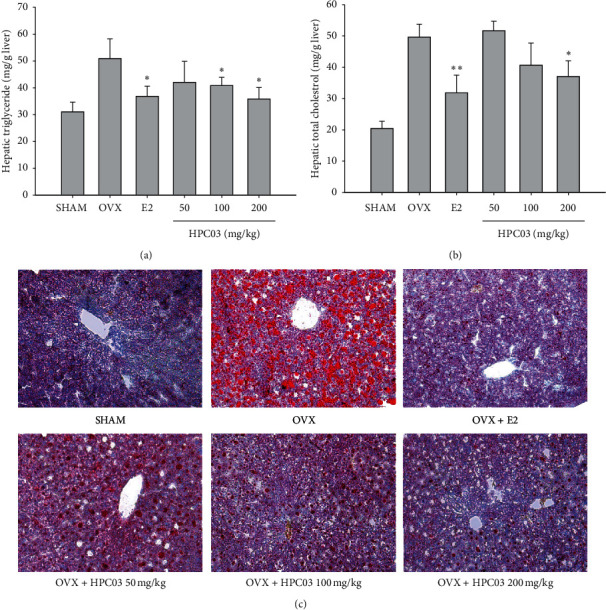
Effect of HPC03 hepatic lipid profiles in ovariectomized rats. At the end of the treatment period, the liver was separated, and then (a) the triglyceride (TG) and (b) total cholesterol (TC) were measured, as described in Materials and Methods. (c) The livers were fixed with 4% formaldehyde and then embedded in frozen section media. Cryosections were stained Oil red O and with hematoxylin examined by light microscope (200x). The results are presented as mean ± SD. Significant differences compared with the OVX group are indicated by ^*∗*^*p* < 0.05 or ^*∗∗*^*p* < 0.01.

TG and TC in livers of the OVX group were 50.9 ± 7.3 mg/g liver and 49.7 ± 4.1 mg/g liver, respectively, which were significantly higher than those in the SHAM groups (*p* < 0.01). In the SHAM group, they were only 31.0 ± 3.6 mg/g liver and 20.4 ± 2.3 mg/g liver. The 200 mg/kg OVX + HPC03 group had a marked effect on controlling liver TG and TC levels, with TG 35.8 ± 4.3 mg/g liver and TC 37.0 ± 5.0 mg/g liver, which were even lower than those of the SHAM group. The OVX + *E*2 group had significantly lower hepatic TG and hepatic total cholesterol levels compared to the OVX group (Figures 2(a)) and 2(b)).

Oil red o staining further confirmed that lipid accumulation was excessive in the livers of the SHAM and OVX groups (Figure 2(c)), whereas it was OVX + *E*2 and OVX + HPC03 groups. These data indicate that estrogen may exert inhibitory effects on hepatic steatosis.

### 3.6. Effect of HPC03 Hepatic Antioxidant Levels

**Figure 3:**
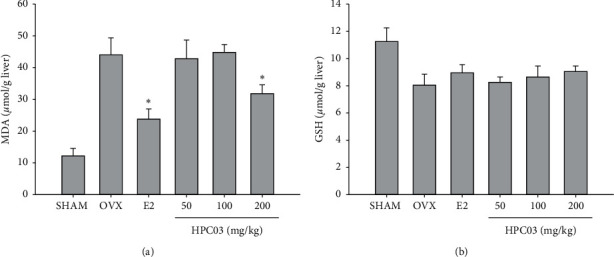
Effect of HPC03 hepatic antioxidant levels in ovariectomized rats. At the end of the treatment period, the liver was separated, and then the MDA and GSH were measured, as described in Materials and Methods. The results are presented as mean ± SD. Significant differences compared with the OVX group are indicated by ^*∗*^*p* < 0.05.

MDA level increased by 256.4%p in the OVX group (44.2 ± 5.4 *μ*mol/g liver) compared with that of the SHAM group (12.4 ± 2.4 *μ*mol/g liver). However, treatment with 200 mg/kg in the OVX + HPC03 group significantly decreased MDA level (32.1 ± 2.8 *μ*mol/g liver; Figure 3(a)). The OVX + *E*2 group had significantly a lower MDA level compared to the OVX group.

The OVX group had significantly reduced 28.3%p GSH levels (8.1 ± 0.8 *μ*mol/g liver) compared with those of the SHAM group (11.3 ± 0.9 *μ*mol/g liver). In the 200 mg/kg OVX + HPC03 group, the GSH was decreased 12.3%p compared to that of the OVX group. However, no significant change in GSH was observed among the treated groups (Figure 3(b)).

## 4. Discussion

Estrogen is mainly produced in the ovaries and is produced by the conversion of androgen in the adrenal glands and fat, and *β*-estradiol is important in hormone regulation. Estrogen deficiency affects the destruction of energy metabolism in women as well as the formation and distribution of adipose tissue [24–27]. Estrogens regulate growth hormone (GH) production and energy homeostasis. For studies related to postmenopausal women, natural menopausal animal models and artificially ovariectomized models are used. Among them, ovariectomized models are more commonly used [28, 29]. Several studies have also shown that estrogen receptor alpha (ER*α*) is associated with weight control and lipid accumulation [7, 30]. As in the study of Palmisano and Koshy et al., fatty liver is induced in ovariectomized rodents, and the improvement effect of fatty liver has been verified when treated with similar estrogens such as estrogen or phytoestrogens [8, 10, 31].

Many researchers have conducted experiments on plant extracts and single compound isolated from extracts using various NAFLD models. Among them, the plant extracts studied were as follows: the lemon balm extract ALS-L1023, Vitex Agnus-Castus L. (Verbenaceae), volatile oil, mulberry leaf, licorice root, *Uncaria tomentosa*, *Rubus crataegifolius* roots, *Lycium barbarum*, and white button mushroom (WBM) [32] [19, 33–38]. Many single compound including flavonoids, alkaloids, polysaccharides, quinones, terpenes, coumarins, lignans, saponins, cardiac glycosides, phenolic acids, and amino acids have been shown to have considerable therapeutic effects on NAFLD [13,15,19,39]. Among them, flavones, flavans, isoflavanes, and coumestans are also used as plant estrogens for the treatment of menopause. These phytoestrogenic activities require interaction with the ERs (ER*α* and ER*β*) [10, 40]. We previously reported that the herbal formula HPC03 increased the cell growth and the estrogen-dependent genes, such as *Psen2, Pgr,* and *Ctsd,* and stimulated proliferation of ER-positive MCF-7 cells. Also, HPC03 inhibited bone resorption and improved bone formation in ovariectomized rats [21].

Many researchers suggested that a crude extract of estrogenic potential might have a protective effect against NAFLD in menopause conditions. In this study, we investigated the NAFLD protection of HPC03, which exhibits estrogen-like action. To evaluate the effect of HPC03 on NAFLD, both an in vitro model of lipid accumulation and an in vivo menopausal model of NAFLD were used.

High concentrations of glucose, BSA, and various fatty acids are used to induce lipid accumulation in HepG2 cells. According to Araya et al. [41], patients with NAFLD have a high content of simple unsaturated fatty acids in the blood and the highest distribution of oleic acid. Based on this, Gomez-Lesson et al. has established a fatty liver induction model by treating oleic acid in HepG2 cells [42]. In addition, when treating HPC03 by inducing lipid accumulation using oleic acid, it was confirmed that fat accumulation was highly dependent and significantly reduced.

HPC03 showed the effect of inhibiting the accumulation of lipids in hepatocytes at nontoxic concentrations. Based on in vitro experimental results, we attempted to confirm the results in an in vivo system of a menopause animal model in which nonalcoholic fatty liver was induced.

The OVX group compared to the SHAM group showed tissue lipid accumulation between body weight and increased abdominal fat, as described in previous other studies [1, 43]. The estrogen treatment with OVX prevented weight gain and increased adiposity. We found that OVX increased fatty acid production and decreased the production and accumulation of fat by inducing fatty acid oxidation in the HPC03 group.

The liver is the main organ in which the synthesis and metabolism of lipoproteins occur. Therefore, if there is a problem with dry health, lipoprotein levels may be higher. TG and TC were measured as indicators related to lipid accumulation in liver tissue. The OVX group had a significant increase compared to the SHAM group, and these results are consistent with the Sock or Chong et al. study [44, 45]. Similar to the results of Chong et al.'s study in which phytoestrogen material was used as a candidate [45], HPC03 was also found to significantly reduce the TG and TC deposited during the experiment periods. According to Liu et al., plasma triglycerides below normal or control levels with severely high liver levels are associated with disturbances in the secretion of newly synthesized hepatic triglycerides, causing fat accumulation in the liver. At 12 weeks after the operation, serum TC of the HPC03 group was not statistically different. However, HPC03 could lower the hepatic TC value to the normal value.

ALT and AST are distributed throughout the body, whereas ALT is mainly present in the cytoplasm of hepatocytes, while AST is distributed in the cytoplasm and the mitochondria of the heart, liver, and kidney. ALT and AST can be easily measured in the blood, and since ALT is a hepatocyte specific enzyme, the elevation of ALT directly reflects hepatocyte damage such as fatty liver, hepatitis, or hepatic necrosis [33]. Fatty livers were shown to develop in the OVX group, and their serum ALT and AST activities were found to increase significantly. This suggested that OVX in rats could induce liver damage, which is in line with other findings in postmenopausal women and OVX animals. HPC03, as shown in the current study, exerted a significant decrease in serum ALT and AST in the OVX rats with HPC03. The results, therefore, suggested that HPC03 has hepatoprotective effects by preventing the liver from having deposits of lipids. HPC03 extract, like consumption of E2, provided prominent protective effects to the liver to overcome the detrimental oxidative stress associated with ovariectomy by decreasing the levels of hepatic MDA and increasing the levels of the hepatic total glutathione.

Menopause can induce abnormal serum lipid production, substantially affecting liver function, causing excessive production of reactive oxygen species (ROS), starting lipid peroxidation, impairing liver function, and adversely affecting the cardiovascular system [2, 8, 29]. Hepatocyte possesses an enzyme system to protect against oxidative stress, and regulating endogenous antioxidants in the liver not only prevents fatty liver production but also prevents the onset of cardiovascular disease. In the present study, we found oxidative stress to be inhibited by the HPC03 as assessed by a decrease in liver MDA levels. Ovariectomized rats exhibited elevated levels of MDA and enzymic antioxidants SOD.

## 5. Conclusion

HPC03 improved lipid profiles (hepatic TG and total cholesterol) and significantly reduced increased blood AST, AST, TG, and total cholesterol. It also induced suppression of lipid peroxides in hepatic damaged by the ovariectomized rat model. In addition to the previously studied estrogen-like effects, an increase in the antioxidant-related indicators of HPC03, especially a decrease in the content of lipid peroxides, was associated with a liver damage as a fatty liver in rats as an animal model for postmenopausal women. Clinical studies are warranted to confirm the beneficial effects of HPC03 in postmenopausal women with NAFLD.

## Figures and Tables

**Table 1:** Effect of HPC03 on food intake, gain in body abdominal fat mass, and liver weight in ovariectomized rats.

Contents/group	SHAM	OVX	E2	HPC03 (mg/kg)		
				50	100	200
Food intake (g/day)	21.3 ± 2.1	26.1 ± 4.03	21.9 ± 6.85	24.7 ± 7.01	23.3 ± 4.83	22.8 ± 5.83
Body weight (g)						
Initial weight	175.0 ± 13.6	188.5 ± 12.0	181.7 ± 17.0	186.6 ± 13.4	186.4 ± 13.8	186.2 ± 1.2
Final weight	322.1 ± 21.5	435 ± 31.7	349.0 ± 24.2	382.0 ± 3.5	372.1 ± 20.9	370.0 ± 2.4
Weight gain	147.0 ± 17.2	246.5 ± 27.4	167.3 ± 19.9^*∗*^	195.4 ± 4.0^*∗*^	185.6 ± 16.6^*∗*^	183.8 ± 6.0^*∗*^
Abdominal fat mass (g)	80.7 ± 5.8	136.5 ± 8.3	96.89 ± 2.8^*∗*^	125.2 ± 8.9	124.8 ± 9.8	107.4 ± 7.8^*∗*^
Relative organ weight (%)						
Liver	2.6 ± 0.3	3.2 ± 0.5	2.7 ± 0.3	3.0 ± 0.8	2.8 ± 0.4	2.9 ± 0.4

The results are presented as mean ± SD. Significant differences compared with the OVX group are indicated by ^*∗*^*p* < 0.05.

**Table 2:** Effect of HPC03 serum transaminase activities and lipid profiles in ovariectomized rats.

Contents/group	SHAM	OVX	E2	HPC03 (mg/kg)		
				50	100	200
ALT (unit/mL)	97.6 ± 15.5	132.0 ± 6.1	105.6 ± 24.0^*∗*^	123.0 ± 4.02^*∗*^	118.2 ± 9.1^*∗*^	76.5 ± 16.3^*∗∗*^
AST (unit/mL)	27.0 ± 4.03	38.0 ± 5.0	28.3 ± 5.0	37.0 ± 2.0	33.0 ± 1.0	31.0 ± 2.0^*∗*^
Triglyceride (mg/dL)	75.0 ± 6.1	166.6 ± 21.5	106.3 ± 18.2^*∗*^	116.4 ± 40.0	120.3 ± 28.0^*∗*^	113.7 ± 17.7^*∗*^
Total cholesterol (mg/dL)	96.3 ± 12.4	145.3 ± 21.5	130.6 ± 17.9	110.8 ± 7.5	121.4 ± 10.0	117.2 ± 22.3

At the end of the treatment period, the blood was collected for serum, and then, the ALT, AST, triglyceride (TG), and total cholesterol (TC) were measured as described in Materials and Methods. The results are presented as mean ± SD. Significant differences compared with the OVX group are indicated by ^*∗*^*p* < 0.05 or ^*∗∗*^*p* < 0.01.

## Data Availability

The data sets used and/or analyzed during the current study are available from the corresponding author upon reasonable request.
